# Reviews

**Published:** 1881-08

**Authors:** 


### Reviews.

**An Introduction to Pathology and Morbid Anatomy.** By F. HENRY GREEN, M. D., Fellow of the College of Physicians, London; Lecturer on Pathology and Morbid Anatomy, at Charing Cross Hospital Medical School, etc., etc. Fourth American from fifth revised and enlarged English edition, with one hundred and thirty-eight fine engravings. Philadelphia: H. C. Lea's Son & Co. 1880.

Those who have studied and worked with Dr. Green's treatise will be glad to get this new edition. It has been carefully revised by the author with the view of making it include the most recent advances in pathology, and we believe that it will now be more popular than ever as a text book. For practical, ordinary daily use, we consider it the best work that is to be had for students of pathology and morbid anatomy.

---

**Cyclopædia of the Practice of Medicine**; edited by Dr. VON ZIEMSEN, Professor of Clinical Medicine in München, Bavaria. Vol. IX. Diseases of the Liver and Portal Vein, with the chapter relating to Insterstitial Pneumonia, by Prof. Ponfick, of Rostock; Prof Thierfelder, of Rostock; Prof. Von-Schneppel, of Tuebingen; Prof. Heeler, of Kiel, and Professors Leichtenstern and Tuergensen, of Tuebingen. New York: Wm. Wood & Co. 1880.

The ninth volume of this great work is fully equal to the other volumes and the completion of the work will be gratifying to every subscriber.

---

**The Principles and Practice of Surgery**; being a Treatise on Surgical Diseases and Injuries. By D. HAYES AGNEW, M. D., Professor of Surgery in the Medical Department of the University of Pennsylvania. Profusely illustrated. Vol. II. Philadelphia: J. B. Lippincott & Co. 1881.

The first volume of this work made its appearance, if we remember right, several years ago, and the work was to be completed in two volumes, but the author has been obliged to add a third. The second volume contains more than a thousand pages and treats in an exhaustive and complete manner of dislocations, diseases of joints, excisions, amputations, diseases of the genito-urinary organs, surgical diseases of women, etc., etc. This work will be of use especially as a book of reference; but, also, as a text book in the medical colleges, especially in the pure surgical subjects; it is unsurpassed in the English literature. We have, indeed, larger works as Holmes' (shortly to be re-edited and revised by an American corpse of surgeons), but we doubt that any single man has undertaken and as successfully carried out his purpose to write a book on general surgery, as has Dr. Agnew.

---

**Hernia, Strangulated and Reducible**; with Cure by Subcutaneous Injection; together with Suggested and Improved Methods for Kelatomy, etc. By JOSEPH H. WARREN, M. D. With illustrations. Boston: Charles N. Thomas, 215 Tremont street. 1881.

This book gives an account of the operation, by subcutaneous injection, for the cure of hernia, as practiced by the late Dr. Heaton, and improved by the author. It is especially as a record of this operation that the work is of interest. Most of the chapters contain scarcely anything which is not to be found in the surgical manuals.

---

**Easy Lessons in Sanitary Science.** By JOSEPH WILSON, M. D., Med. Director U. S. Navy. Philadelphia: Presley Blackiston 1881.

This little book gives some needed lessons specially on the subject of drainage. It is written in so pleasant a style, and free from all technicalities that it may well be entitled *Easy Lessons*—but as there is no royal road to learning, the greatest success which we can wish for the book, is, that its perusal may put ideas into the heads of some, who are in blissful ignorance of their violations of sanitary laws, so that they may either study proper works, or consult those who have made a study of the broad subject of hygiene and thus be able intelligently to correct dangerous surroundings of their homes.

